# Genome editing approaches to harness cytokinin–salicylic acid crosstalk for plant protection

**DOI:** 10.3389/fgeed.2025.1687599

**Published:** 2025-10-20

**Authors:** Muhammad Naseem, Khalid Muhammad

**Affiliations:** ^1^ Department of Environmental Science and Sustainability, College of Natural and Health Sciences, Zayed University, Abu Dhabi, United Arab Emirates; ^2^ Department of Bioinformatics, Biozentrum Am Hubland, University of Würzburg, Würzburg, Germany; ^3^ Department of Biology, College of Science, UAE University, Al Ain, United Arab Emirates

**Keywords:** genome editing, cytokinin signalling, plant immunity, growth-defence trade-off, two-component system

## 1 Overview on cytokinin signalling and metabolism in plants

Cytokinins (CKs) are a class of adenine-derived small-molecule compounds that regulate the entire bauplan of plants. Central to CK perception is the Two-Component Signalling (TCS) system, a signal transduction mechanism conserved from prokaryotes and only adapted in plants among the eukaryotes. In *Arabidopsis thaliana*, CK perception is initiated at the plasma membrane by histidine kinase receptors (AHK2, AHK3, and AHK4/CRE1), which contain a CHASE (Cyclases/Histidine kinases Associated Sensory Extracellular) domain. This phosphorelay occurs via Arabidopsis Histidine Phosphotransfer Proteins (AHPs) to nuclear-localized Response Regulators (ARRs), which then regulate the transcription of cytokinin-inducible genes (Zhao et al., 2024). The ARRs are categorized into two types: type-B ARRs (e.g., ARR2, ARR10, ARR12) act as positive transcriptional activators, while type-A ARRs (e.g., ARR5, ARR6, ARR7, ARR15) serve as negative regulators that dampen CK signalling. The phosphorelay cascade thus allows for subtle regulation of CK output by cues orchestrated by developmental as well as biotic and abiotic stress conditions (Argueso et al., 2009).

The predominant CKs forms in plants include isoprenoid and aromatic species, with the former being the most abundant. CK biosynthesis begins with the enzyme adenylate isopentenyltransferase (IPT), which catalyzes the transfer of an isopentenyl group from dimethylallyl diphosphate (DMAPP) to adenosine monophosphate (AMP), forming isopentenyladenine ribotides (iP-ribotides) (Kakimoto, 2001). In *Arabidopsis*, both ATP/ADP-IPTs and tRNA-IPTs exist, with the former primarily involved in active CK biosynthesis and the latter producing *cis*-zeatin-type CKs. The hydroxylation of iP-ribotides by cytochrome P450 monooxygenases (CYP735A1/A2) converts them into *trans*-zeatin ribotides, which are precursors of *trans*-zeatin (Takei et al., 2004). These nucleotides are then converted to free bases by Lonely Guy (LOG) enzymes, which directly produce the biologically active forms of CKs through a single-step phosphoribohydrolase reaction (Kurakawa et al., 2007). CK deactivation primarily occurs through irreversible degradation by cytokinin oxidase/dehydrogenase (CKX) enzymes, which cleave the N^6^ side chain of isoprenoid CKs, effectively reducing their bioactivity (Werner et al., 2003). In contrast, reversible deactivation involves glycosylation, in which CKs are conjugated with glucose to form O- or N-glucosides. These conjugated forms serve as storage or transport forms and can be reactivated under specific physiological conditions (Hou et al., 2004). This dynamic interplay of biosynthesis, activation, and degradation ensures precise regulation of CK levels, maintaining hormone homeostasis across developmental stages and environmental contexts. The broader crosstalk potential of CK signalling proteins (Naseem et al., 2012; Naseem et al., 2014) within the plant interactome offers key targets for genome editing, enabling precise biotechnological modulation to safeguard plant health against diverse pathogens.

## 2 Cytokinin-mediated immune defense networks in plants

Plants, being sessile organisms, rely on intricate hormonal networks to coordinate growth, development, and immunity in response to an ever-changing environment. Traditionally viewed through the lens of development, CK has more recently been identified as a crucial regulator of plant immunity, exhibiting either positive or negative effects during plant-pathogen interactions. Depending on the type of pathogen and the context of infection, CK responses either promote or suppress infection of the host plant (Naseem and Dandekar, 2012). One of the most intricate aspects of this regulatory complexity is the interplay between growth-promoting and defence-related hormones. Among these, CKs and salicylic acid (SA) stand out as central players in modulating plant immunity, particularly in response to biotrophic and hemi-biotrophic pathogens. The interaction between these signaling pathways is not linear but is orchestrated through TCS-signaling, feedback loops, and concentration-dependent mechanisms that determine the growth-defense trade-off (Choi et al., 2010; Naseem et al., 2012; Argueso et al., 2012; Gupta et al., 2023). Likewise, the interaction between CK and Jasmonate-mediated signaling also modulates immune responses in plants (Naseem et al., 2013). While widely studied in growth and development, the antagonistic interaction between CK and auxin also impacts immune responses in plants (Naseem and Dandekar, 2012). In the following, we provide an overview of the immune-related processes that are directly or indirectly influenced by CK in plants.

### 2.1 A functional and molecular crosstalk between cytokinin and salicylic acid

The interplay between CK signaling and SA-mediated immunity is multifaceted and has been a focal point of research in plant immunity. SA, a phenolic compound synthesized via the isochorismate pathway, is indispensable for both systemic acquired resistance (SAR) and local resistance against biotrophic and hemibiotrophic pathogens (Vlot et al., 2009). Its downstream signaling is largely mediated by *NPR1*, *nonexpressor of PR Genes 1 (NPR1)*, and the induction of *pathogenesis-related* protein (*PR) genes*, particularly *PR1*, which serves as a molecular marker for SA-based immunity in plants (Dong, 2004; Pieterse et al., 2009). CK can potentiate SA signaling at multiple regulatory nodes. It promotes *PR1* gene expression through activation of type-B ARRs (Choi et al., 2010). Specifically, ARR2 has been shown to interact with TGA3, a transcription factor central to the SA pathway, thereby directly linking CK perception to SA-mediated transcriptional responses (Choi et al., 2010; Argueso et al., 2012). Similarly, exogenous application of CK or transgenic overproduction of CK enhances resistance to *Pseudomonas syringae* by upregulating SA-responsive genes (Naseem et al., 2012; 2013; Choi et al., 2010).

### 2.2 Negative regulation by Type-A ARRs and growth-defense trade-off

Conversely, SA can suppress CK signaling downstream of the SA accumulation (Argueso et al., 2012). This reciprocal regulation establishes a feedback loop that fine-tunes immune responses, helping to balance effective pathogen defense with the metabolic costs associated with sustained immune activation. While type-B ARRs act as defense promoters, type-A ARRs limit SA-induced gene expression, acting as a critical buffer to prevent overactivation. Thus, the type-A ARR genes are rapidly upregulated during immune responses, forming a negative feedback loop to switch off CK-enhanced SA signaling once the threat subsides. Functional studies using *arr*-mutants have shown enhanced SA responses and plant resistance to pathogen, confirming the suppressive role of type-A ARRs in plant immune defense. It is noteworthy to mention that the target of the A-type ARRs is part of the SA-responsive signaling pathway and that its position is downstream of SA (Argueso et al., 2012). These regulators may function by sequestering AHPs or directly competing with type-B ARRs for phosphorylation, thus fine-tuning immune activation.

### 2.3 Pathogen-specific hormonal responses and the dual role of cytokinin in pathogen defense

The functional output of CK-SA crosstalk is not uniform across pathogen types. Biotrophic pathogens, which feed on living tissue, are effectively deterred by SA-dependent responses. In contrast, necrotrophic pathogens, which kill host cells, often require jasmonic acid (JA) and ethylene (ET) for effective resistance (Pieterse et al., 2009). Interestingly, certain pathogens secrete CK to manipulate host TCS signaling. *Agrobacterium tumefaciens*, for instance, uses tumor-inducing (Ti) plasmids that encode CK biosynthesis genes to promote host cell proliferation and suppress immunity (Veselova et al., 2021). In such cases, host manipulation of type-A ARR expression might offer a route to restore immunity and disrupt pathogen benefit. Recently, Gupta et al. (2020) demonstrated that both endogenous and exogenous CK treatments in tomato trigger systemic immunity and enhance resistance against *Botrytis cinerea* and *Oidium neolycopersici*. This immune activation is mediated via SA and ethylene-dependent signaling pathways and involves modulation of the pattern recognition receptor (PRR) LeEIX2 trafficking, a critical step in immune perception and signaling. Importantly, the presence of functional CK perception machinery within the host was shown to be essential for this protective effect, highlighting CK’s role as a potent defense-priming molecule (Gupta et al., 2020).

In a subsequent study, Gupta et al. (2021) provided evidence that CK also exerts a direct inhibitory effect on the fungal pathogen itself. Treatment with CK impaired *B. cinerea* development and virulence by disrupting cytoskeleton organization, endocytosis, cell cycle progression, and intracellular trafficking processes. These disruptions ultimately reduce fungal growth and pathogenesis. Furthermore, CK treatment inhibits sporulation, spore germination, and lesion development on infected plant tissue, indicating a dual mode of action, thus affecting both host resistance and pathogen viability (Gupta et al., 2021; Gupta et al., 2023).

### 2.4 Stomatal immunity, reactive oxygen species (ROS) and cytokinin

In addition to transcriptional regulation, CK contributes to rapid, non-transcriptional defense responses during the earliest stages of pathogen attack. A key component of this pre-invasive immunity is stomatal closure, which prevents bacterial entry after guard cells perceive pathogen-associated molecular patterns (PAMPs) such as flg22 (Melotto et al., 2006). CK enhances this process by stimulating a reactive oxygen species (ROS) burst in the apoplast. This occurs through the induction of peroxidase activity (Arnaud et al., 2017) and the regulation of NADPH oxidase function, leading to robust ROS accumulation (Arnaud et al., 2017; Naseem et al., 2012). These rapid oxidative signals act as early antimicrobial barriers, strengthening basal immunity at the site of infection.

Crucially, these fast, non-transcriptional defenses operate in parallel with slower, transcription-dependent immune gene activation, thereby establishing multi-layered protection against pathogens (Torres and Dangl, 2005; Lu et al., 2010). By influencing both early recognition events mediated by pattern recognition receptors (PRRs) such as FLS2 and downstream signaling cascades, CK emerges as an important regulator of basal and innate plant immunity.

## 3 Update on CRISPR/cas-based editing of cytokinin pathway genes in plants

Understanding CK–SA crosstalk opens the door to precision genetic engineering aimed at rebalancing the growth-defense trade-off. The CRISPR/Cas system allows for specific, efficient, and heritable edits in plant genomes, offering new possibilities to modulate key regulators of CK signalling. Here, we highlight recent advances in genome editing of CK pathway genes and their impacts across diverse plant species. Several studies illustrate how targeted modification of CK-related genes can reprogram developmental and stress-response pathways in crops, offering new strategies to enhance resilience and productivity.

For instance, in tomato, CRISPR-mediated mutation of *SlHP2* and *SlHP3*, upstream phosphotransfer proteins, reduced stomatal density, improved water retention, and decreased oxidative damage under drought stress, demonstrating that modification of upstream CK signalling enhances stress resilience (Vorlop et al., 2023). In rice and barley, editing of *CKX* genes altered root architecture, enhanced seed biofortification (e.g., increased Zn accumulation), and improved drought tolerance without yield penalties, thus validating CK modulation via genome editing as both practical and agronomically beneficial (Joshi et al., 2018; Holubová et al., 2018). In *Jatropha curcas*, knockout of *CYP735A*, a CK biosynthesis enzyme, reduced CK levels and severely stunted growth, reinforcing the importance of subtle editing strategies (Gu et al., 2020). These examples highlight the value and complexity of manipulating CK pathways, while elevated CK can promote yield and resilience in certain contexts. Likewise, Xing X. et al. (2025) demonstrated that *BvHP4b*, a histidine phosphotransfer protein in sugar beet, is upregulated by CK and localizes to the cell membrane. Using CRISPR/Cas9, *bvhp4b knockout* plants exhibited increased susceptibility to *Pseudomonas syringae*, whereas *BvHP4b* overexpression enhanced taproot growth and disease resistance by regulating immunity and SA synthesis (Xing et al., 2025b). Furthermore, BvHP4b interacts with BvCDC2, acting as a positive regulator of both development and defense in sugar beet. These recent examples underscore the potential of genome-editing strategies in modulating CK responses and pave the way for identifying more systematic targets to fine-tune immune defense in plants.

## 4 Proposed genome editing strategies of the cytokinin pathway to modulate plant immunity

CK metabolism and signaling provide powerful entry points for crop improvement, offering leverage over plant architecture, reproductive output, nutrient allocation, and stress resilience. However, classical genetic studies have shown that blunt manipulations, for instance, complete knockouts or constitutive overexpression, cause severe pleiotropy and developmental disruption. For instance, *Arabidopsis cytokinin receptor* mutants display dramatic developmental defects such as altered shoot–root balance, reduced meristem activity, and compromised fertility (Higuchi et al., 2004; Nishimura et al., 2004; Riefler et al., 2006). Similar problems have been documented for mutants in *AHP* genes and *ARR* regulators (To and Kieber, 2008). Likewise, the functional redundancy that has been observed among the different members of each gene family erases the effects of a single mutation, for example, in a single A-type *ARR-A* gene mutant, other A-type *ARR* genes will function and compensate the effect owing to genetic redundancy. Likewise, functional redundancy has also been observed among *AHK, AHP*, and *ARR-B* genes as well. These findings underscore why CK pathways remain underutilized in applied breeding, as their pleiotropic roles in both growth and immunity demand very precise regulation.

We propose strategies how CRISPR-based tools, such as base and prime editing, cis-regulatory editing, and tissue-specific dCas9 modulation, can convert cytokinin pathway genes from blunt levers into tuneable switches (Table 1). Importantly, such precision editing is not only about safeguarding developmental stability but also about enhancing resistance to major pathogens while maintaining yield. At the level of perception, cytokinin receptors (AHKs) act as critical protein hubs for integrating growth and immunity. CKs have been shown to potentiate SA-dependent defense while sometimes antagonizing jasmonic acid (JA)-mediated responses (Choi et al., 2010; Argueso et al., 2012). In Arabidopsis, elevated CK signaling boosts immunity against *Pseudomonas syringae*, a hemibiotrophic bacterial pathogen that suppresses SA pathways (Choi et al., 2010). Editing AHKs to create hypomorphic alleles holds promise to maintain sufficient defense activation without the severe developmental collapse observed in null mutants (Table 1).

**TABLE 1:** Proposed genome editing strategies and target sites in cytokinin signaling and metabolic pathways in plants.

Target gene/class	Editing strategy	Rationale	Risk mitigation	Infection context
Cytokinin receptor genes (AHKs)	Base/prime editing of receptor domains to generate hypomorphic alleles	Modulates cytokinin sensitivity rather than abolishes signaling	Avoids severe developmental defects reported in null mutants (Higuchi et al., 2004; Riefler et al., 2006)	*Pseudomonas syringae* (Arabidopsis): enhanced resistance via cytokinin–SA synergy (Choi et al., 2010; Naseem et al., 2017)
Histidine phosphotransfer proteins (AHPs)	Cis-regulatory editing of promoter elements	Subtly alters expression levels in specific tissues	Prevents systemic disruption of signaling cascade	Broad immune tuning; balance between SA/JA defenses (Cortleven et al., 2019)
Type-A ARRs (Negative regulators)	Promoter editing to enhance expression in stress-related tissues	Fine-tunes cytokinin signaling under stress	Reduces pleiotropy by restricting changes to stress-responsive organs (To and Kieber, 2008)	Limits overactivation of cytokinin immunity to reduce growth penalties under chronic infection
Type-B ARRs (transcriptional activators)	Tissue-specific dCas9-based activation or repression	Targets shoot meristems or reproductive tissues selectively	Maintains normal cytokinin signaling in other organs (To and Kieber, 2008)	*Xanthomonas oryzae* (rice): ARR-like TFs linked to leaf immunity
Downstream cytokinin-responsive genes	Allelic engineering of effector genes (e.g., senescence or nutrient transport)	Redirects cytokinin outputs toward specific developmental/agronomic traits	Avoids manipulation at the master-regulator level	PR genes induced by cytokinin; resistance to *Clavibacter michiganensis* (tomato)
IPTs (isopentenyl transferases)	Cis-regulatory editing or tissue-specific activation	Fine-tunes cytokinin biosynthesis in key organs (e.g., roots, reproductive tissues)	Prevents excessive cytokinin accumulation and maintains systemic balance only under pathogen attack	*Fusarium oxysporum* (roots) and *Botrytis cinerea* (flowers): IPT upregulation enhances local defense
CKXs (cytokinin oxidase/dehydrogenases)	Promoter editing or allele engineering for partial activity reduction	Enhances cytokinin levels to improve yield traits (grain number, size)	Avoids severe developmental trade-offs observed in full knockouts; enables tissue-specific modulation	*Magnaporthe oryzae* (rice blast): reduced CKX enhances yield and resistance
LOGs (Lonely Guy family)	Cis-regulatory editing for spatiotemporal control of cytokinin	Restricts cytokinin activation to tissues where it benefits development	Minimizes unintended effects by avoiding systemic increases in active cytokinin levels	Local activation enhances resistance to biotrophic pathogens (Kurakawa et al., 2007)

Likewise, AHPs and ARRs represent downstream nodes where immune trade-offs can be tuned. Type-A ARRs act as negative regulators; promoter editing to increase their expression in stress-responsive tissues holds the potential to help maintain CK homeostasis during pathogen attack by minimizing growth penalties while still enabling timely defense induction (To and Kieber, 2008; Cortleven et al., 2019). In contrast, type-B ARRs activate transcriptional programs that enhance SA-mediated immunity. In rice, ARR-like transcription factors are linked to defense against *Xanthomonas oryzae pv. oryzae* (bacterial blight) (Jiang et al., 2013). Tissue-specific dCas9-based modulation of type-B ARR activity could boost resistance in leaves or reproductive tissues, while sparing roots and vegetative organs where CK regulation is critical for growth.

Downstream CK-responsive target genes provide another means of channelling defense responses in plants. CK upregulates *PR* genes and nutrient transporters, many of which are directly implicated in immunity (Choi et al., 2010). For example, in tomato, cytokinin-induced PR gene expression enhances resistance to *Clavibacter michiganensis*, the causal agent of bacterial canker (Giron et al., 2013). Allelic engineering of such a target gene could tailor CK outputs to reinforce barrier functions or sustain SA-dependent resistance.

The metabolic control points of CK biosynthesis and degradation (Mok and Mok 2001) strongly influence immunity. *IPT* genes, which catalyze the rate-limiting step of CK biosynthesis, are natural candidates for targeted editing. Restricting *IPT* activation to roots via cis-regulatory editing could enhance resistance to soil-borne pathogens such as *Fusarium oxysporum* (O'Brien and Benková, 2013) while avoiding systemic CK overload. CKs are likely transported to distal organs via the xylem, predominantly in the form of trans-zeatin riboside (tZR). The deployment of pathogen-inducible IPT gene expression systems (Naseem et al., 2017) offers a targeted strategy to achieve localized, demand-driven CK biosynthesis at infection sites, thereby restricting systemic outflux and maintaining hormonal balance. Similarly, *IPT* upregulation in reproductive tissues could strengthen floral defense against necrotrophs like *Botrytis cinerea* (Choi et al., 2010), which colonizes flowers and fruits.

On the degradation side, *CKX* genes are well known for their impact on yield traits such as grain number in cereals (Ashikari et al., 2005). CKX activity also constrains immunity by lowering cytokinin availability. Partial suppression of *CKX* in rice has been associated with both improved grain productivity and stronger resistance to *Magnaporthe oryzae* (rice blast) (Yuan et al., 2020). Precision promoter editing or allele engineering could sustain higher basal CK to prime defense without the developmental liabilities caused by complete knockouts.

The LOG (Lonely Guy) family, which activates CKs by converting nucleotides into bioactive forms, also intersects directly with pathogen responses. LOG activity at infection sites can create localized CKs bursts, enhancing SA signaling and resistance to biotrophic pathogens (Kurakawa et al., 2007; Argueso et al., 2012). For example, *LOG* expression in rice inflorescences supports reproductive success, but its immune role suggests that restricting *LOG* activation to pathogen-exposed tissues (e.g., young leaves vulnerable to *M. oryzae*) could fortify local resistance.

Altogether, these strategies underscore CK’s dual role in development and immunity. With CRISPR technologies, CK genes can be engineered to provide fine-tuned modulation rather than binary on/off changes. By coupling promoter editing, hypomorphic alleles, and tissue-specific modulation, breeders can potentially unlock CK’s pathway for disease resistance against pathogens like *P. syringae, X. oryzae, M. oryzae*, *F. oxysporum*, and *C. michiganensis* without compromising yield stability. The broader implication is that CK editing must be framed within the growth–defense trade-off. As a hormone that integrates developmental and immune networks, CK cannot be manipulated for productivity alone. The proposed precision editing strategies (Table 1) may enable CK pathway genes to act as adjustable valves, balancing resource allocation between defense and yield. This may offer a paradigm shift from viewing CK as a source of uncontrollable pleiotropy to treating it as a versatile tool for next-generation crop resilience.

## 5 Future outlook

CRISPR/Cas has transformed plant genome editing, offering unprecedented precision for crop improvement, but several obstacles limit its widespread application. Current delivery methods, including agrobacterium-mediated transformation and biolistics, often require labor-intensive tissue culture and regeneration protocols that vary across genotypes. DNA repair further constrains outcomes: plants preferentially employ error-prone non-homologous end joining (NHEJ) over homology-directed repair (HDR), reducing the efficiency of precise edits. Although base and prime editors circumvent some of these limitations, concerns about off-target activity remain, necessitating careful guide RNA design and genome-wide validation. Regulatory restrictions add another barrier; for example, the European Union classifies CRISPR-edited plants as GMOs, complicating commercialization and deployment.

Innovations are beginning to mitigate these challenges. Viral-mediated in planta delivery, synthetic biology circuits, and next-generation base editors reduce dependence on tissue culture while expanding editing versatility. Beyond technical advances, CRISPR offers opportunities to reprogram complex hormonal crosstalk, such as between CK and SA. This interaction exemplifies the integration of growth and immunity, with type-A and type-B ARRs acting as molecular switches that hold promise to translate CK signals into pathogen-specific responses. Their regulation not only drives transcriptional reprogramming under stress but also modulates physiological defenses, including stomatal closure and ROS production. The modularity of the TCS, combined with its integration into immune signaling networks, makes CK pathways attractive targets for genome editing.

As climate change intensifies both biotic and abiotic stresses, engineering CK–SA crosstalk represents a promising strategy to balance defense with productivity. Future research should test CK-based defenses under field conditions, explore the conservation of ARR functions across species, and evaluate the long-term fitness consequences of engineered hormonal circuits. Harnessing CRISPR to fine-tune these networks may ultimately yield resilient, resource-efficient crops for sustainable agriculture.
